# Transactivation of G protein-coupled receptors (GPCRs) and receptor tyrosine kinases (RTKs): Recent insights using luminescence and fluorescence technologies

**DOI:** 10.1016/j.coemr.2020.10.003

**Published:** 2021-02

**Authors:** Laura E. Kilpatrick, Stephen J. Hill

**Affiliations:** 1 Division of Bimolecular Sciences and Medicinal Chemistry, Biodiscovery Institute, School of Pharmacy, University of Nottingham, Nottingham, NG7 2RD, UK; 2 Division of Physiology, Pharmacology and Neuroscience, School of Life Sciences, University of Nottingham, Nottingham, NG7 2UH, UK; 3 Centre of Membrane Proteins and Receptors (COMPARE), University of Birmingham and University of Nottingham, The Midlands, NG7 2UH, UK

**Keywords:** G protein-coupled receptor, Receptor tyrosine kinase, Transactivation, Resonance energy transfer, Endocytosis, Oligomeric complexes, 5-hydroxytryptamine receptor 1A, (5-HT1A), adrenoceptors, (AR), bioluminescence resonance energy transfer, (BRET), cannabinoid receptor 2, (CB2R), disintegrin and metalloproteinases, (ADAMs), epidermal growth factor, (EGF), epidermal growth factor receptor, (EGFR), fibroblast growth factor receptor, (FGFR), fluorescence correlation spectroscopy, (FCS), formyl peptide receptor, (FPR), Förster Resonance Energy Transfer, (FRET), free fatty acid, (FFA), G protein-coupled receptors, (GPCRs), GPCR kinases, (GRKs), heparin binding EGF, (Hb-EGF), hepatocyte growth factor, (HGF), human umbilical vein endothelial cells, (HUVECs), insulin growth factor receptor-1, (IGFR-1), insulin receptor, (IR), lysophosphatidic acid receptor 1, (LPA), matrix metalloproteinases, (MMPs), platelet-derived growth factor receptor, (PDGFR), proximity ligation assay, (PLA), reactive oxygen species, (ROS), receptor tyrosine kinases, (RTKs), sphingosine-1-phosphate receptor, (S1PR), tetrahydrocannabinol, (THC), total internal reflection fluorescence microscopy, (TIRF-M), vascular endothelial growth factor, (VEGF), vascular endothelial growth factor receptor 2, (VEGFR2), vasopressin 2 receptor, (V2R)

## Abstract

Alterations in signalling due to bidirectional transactivation of G protein-coupled receptor (GPCRs) and receptor tyrosine kinases (RTKs) are well established. Transactivation significantly diversifies signalling networks within a cell and has been implicated in promoting both advantageous and disadvantageous physiological and pathophysiological outcomes, making the GPCR/RTK interactions attractive new targets for drug discovery programmes. Transactivation has been observed for a plethora of receptor pairings in multiple cell types; however, the precise molecular mechanisms and signalling effectors involved can vary with receptor pairings and cell type. This short review will discuss the recent applications of proximity-based assays, such as resonance energy transfer and fluorescence-based imaging in investigating the dynamics of GPCR/RTK complex formation, subsequent effector protein recruitment and the cellular locations of complexes in living cells.

## Transactivation: a mechanism to increase the signalling diversity of activated G protein-coupled receptors and receptor tyrosine kinases

G protein-coupled receptors (GPCRs) and receptor tyrosine kinases (RTKs) are major classes of cell surface receptors extensively targeted in drug discovery programmes due to their critical roles in health and disease. GPCRs are seven transmembrane spanning receptors that bind a structurally diverse range of ligands [1]. Activation stabilises GPCR conformations favouring downstream signalling via heterotrimeric G proteins (Gα and Gβγ subunits). Four main classes of G proteins exist: G_s_, G_i/o_, G_q/11_, G_12/13_ that direct signalling via distinct effector proteins such as adenylyl cyclase, phospholipase C and Rho GTPases. GPCR activation also promotes the recruitment of GPCR kinases (GRKs) that phosphorylate the GPCR C terminus. This in turn enhances recruitment of β-arrestin which uncouples GPCR/G protein complexes, promoting GPCR desensitisation and endocytosis in addition to G protein-independent signalling pathways; however, the functional significance of this in physiology has been debated [2]. RTKs typically consist of a large extracellular ligand binding domain, a transmembrane domain and an intracellular catalytic kinase domain. RTKs notably bind growth factors such as epidermal growth factor (EGF) and vascular endothelial growth factor (VEGF). Ligand binding typically induces dimerisation of receptor monomers triggering trans-autophosphorylation of C terminal tyrosine residues that act as recruitment sites for intracellular adaptor proteins, such as Src, phosphoinositide 3-kinases (PI3K) and phospholipase C (PLC). These adaptors can themselves be phosphorylated due to the intrinsic kinase activity of RTKs, increasing and diversifying the network of signalling pathways available from activation of a single receptor. RTK-mediated signalling is typically responsible for driving cell proliferation, migration and survival via extracellular signal-regulated kinases 1/2 (ERK1/2), focal adhesion kinase (FAK) and protein kinase A/Akt mediators [3].

GPCRs and RTKs were believed to act as independent signalling entities, until seminal work by Ullrich and colleagues [4] revealed rapid tyrosine phosphorylation of the epidermal growth factor receptor (EGFR) in response to known GPCR agonists. This phenomenon, termed transactivation, is characterized by altered RTK activation and downstream signalling directly attributable to GPCR/RTK interactions. Transactivation offers a mechanism to increase the number and breadth of signalling networks available within each cell, by integrating the diversity of GPCRs and GPCR ligands with the vast signalling networks mediated by activated RTKs [5].

Transactivation of the EGFR has been observed with Class A and Class B GPCR partners including but not limited to the β1, β2 and α1-adrenoceptors (AR), adenosine A1 and A3 receptors, μ opioid receptor, muscarinic M1 and the AT_1_R angiotensin receptor [reviewed in 6]. Evidence of transactivation has been observed in a range of cell types for other RTK family members, such as the vascular endothelial growth factor receptors (VEGFRs), fibroblast growth factor receptors (FGFRs), platelet-derived growth factor receptor (PDGFR), insulin-like growth factor receptor-1 (IGFR-1) and the insulin receptor (IR) [7]. Recent reviews have extensively covered the beneficial roles of transactivation in regulating cardioprotection [6] and vital central nervous system functions [3]. However, disadvantageous signalling as a consequence of transactivation has been identified such as progression from acute to chronic pain (μ opioid/EGFR in opioid-induced hyperalgesia [8]), proliferation of human hyperplastic prostatic cells (α1-AR/EGFR [9]), gastric cancer cell migration (CXCR4/EGFR [10]), poor patient prognosis and increased lymphatic spread in HER2+ breast cancer patients (cannabinoid receptor 2 (CB2R/HER2 [11]) and underlying tumour re-occurrence following anti-VEGF/VEGFR2 therapeutics (sphingosine 1-phosphate receptor/VEGFR2 [12]). In this short review, we focus on recent examples revealing new insights into the molecular mechanisms involved, and highlight some of the new technologies, beyond traditional biochemical techniques, used to investigate transactivation.

## Ligand-dependent and independent mechanisms of transactivation

**Figure 1:**
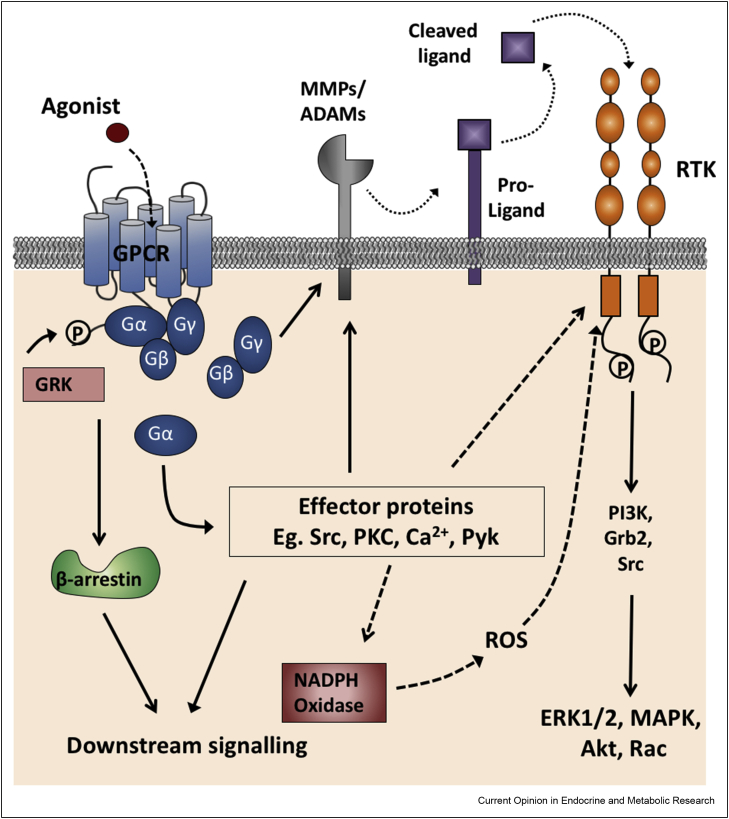
**Ligand-dependent and independent transactivation mechanisms.** GPCRs are seven transmembrane spanning receptors that are activated by agonist binding. This stabilises GPCR conformations favouring the activation and subsequent dissociation of heterotrimeric G proteins (Gα and Gβγ subunits). Gα can mediate signalling via effector proteins such as Src and PKC. In ligand-dependent transactivation, effector proteins activated by GPCR signalling such as Src can themselves induce the activation of matrix metalloproteinases (MMPs) or a disintegrin and metalloproteinases (ADAMs). Evidence also exists suggesting Gβγ subunits may activate MMPs. MMPs and ADAMs cleave pro-forms of RTK ligands that are bound to components of the extracellular matrix (dotted lines). These pro-ligands are then free to diffuse to bind to their cognate RTK. A stylised version of a RTK structure is shown here. RTK ligand binding activates the receptor, triggering monomer dimerisation, auto-transphosphorylation and subsequent downstream signalling pathways. Transactivation can also occur through ligand-independent mechanisms (*dashed arrows*). Effector proteins activated following GPCR activation, such as Src, PKC and Pyk can directly activate RTKs via phosphorylation of tyrosine residues in the C terminus. Additionally secondary messenger molecules generated via effector protein mediated signalling (e.g. reactive oxygen species (ROS) produced by NADPH oxidase) can also mediate direct activation of RTKs.

RTKs can be activated by GPCRs in a ligand-dependent or independent manner (Figure 1). Ligand-dependent transactivation occurs via matrix metalloproteinases (MMPs) or a disintegrin and metalloproteinases (ADAMs) and has been extensively characterised for the EGFR [5]. MMPs or ADAMs cleave RTK pro-ligands bound to extracellular matrix components such as heparin binding EGF (Hb-EGF). These cleaved ligands then bind to cell surface RTKs triggering downstream signalling. Activation of MMPs or ADAMs occurs as a consequence of GPCR activation; however, the exact mechanisms are not fully known but are proposed to involve G_βγ_ subunits [13] or Src [14, 15, 16].

As GPCRs lack intrinsic tyrosine kinase activity, ligand-bound GPCRs indirectly activate RTKs via intracellular protein kinases such as Src, PI3K and Pyk [3]. These effector proteins directly induce RTK activation via phosphorylation of tyrosine or serine/threonine residues. For example, Src-mediated phosphorylation of EGFR has been observed following activation of the corticotropin releasing factor receptor 1 (CRF_1_R) with this transactivation critical for CRF stimulated ERK1/2 signalling [17]. Mediators can also play simultaneous roles in direct RTK activation and transactivation (e.g. Src at CXCR4 and EGFR [15]).

Another mechanism of transactivation is via production of second messengers, notably reactive oxygen species (ROS). NADPH Oxidase produced ROS has been shown to mediate transactivation between formyl peptide receptor 1 (FPR1) with VEGFR2 [18], EGFR [19] and TrkA [20] as well as the formyl peptide receptor 2 (FPR2) with HGF [21]. The multiple RTKs activated by ROS have led to speculation that ROS may be a mechanism for global transactivation [5] supported by recent evidence of ROS-mediated dual transactivation of EGFR and HER2 by neurotensin 1 receptors [22].

Though currently less extensively observed, transactivation can be bidirectional. For example, the lysophosphatidic acid receptor 1 (LPA) and EGFR can reciprocally transactivate and induce proliferation of prostate cancer cells [23]. However, this same positive crosstalk can be suppressed by activation of another GPCR, the free fatty acid receptor (FFA4 [23]). Formation of RTK/GPCR complexes can also alter effector protein coupling to the GPCR partner as seen for the CB2R. In response to tetrahydrocannabinol (THC), the CB2R typically couples to G_q/11_; however, when complexed with HER2, CB2R coupling switches to G_i_ or G_z_ subtypes [11]. This suggests HER2/CB2R is a unique pharmacological entity compared to CB2R that promotes pro-tumoural signalling. RTK-mediated GPCR transactivation is typically more complex than GPCR/RTK and can include tyrosine phosphorylation of GPCRs and GRKs by RTKs or modulation of GPCR serine/threonine phosphorylation by protein kinases (reviewed in Ref. [24]). A recent example of this is internalised EGFR (induced by EGF), which can indirectly mediate inhibition of dopamine D3 receptor signalling by promoting tyrosine phosphorylation of GRK2, subsequently inhibiting D3 signalling, endocytosis and degradation [25]. To add further complexity, different RTKs can induce phosphorylation of the same GPCR but at differing residues (in this instance tyrosines), as observed at the β_2_-AR following ligand activation of the IR [26] or IGF-1R [27].

Challenges remain in unravelling signalling directly attributable to transactivation alone, complicated by RTKs also utilising ‘classical’ GPCR signalling mediators such as G proteins, β-arrestins and GRKs (reviewed in Ref. [7]). For example, G_αi_ is critical to VEGFR2 clathrin-mediated endocytosis, with knockout of G_αi_ retaining VEGFR2 at the cell surface and decreasing downstream VEGFR2 driven signalling [28]. G_βγ_, in conjunction with Src, is implicated in regulating EGFR endocytosis, and mediating interaction of internalised EGFR/Src/GRK complexes [25]. GRKs can also directly regulate RTK-driven signalling (seen for the IGF-1R), with different GRK subtypes exhibiting opposing effects at the same receptor [29], potentially by modulating changes in the lifespan of β-arrestin association. Interestingly, following transactivation (Src and MMP dependent) of the IGF-1R by the vasopressin 2 receptor (V2R), it is the engagement of β-arrestin with IGF-1R and not V2R that is critical for vasopressin stimulated ERK1/2 signalling [16], with suggestions that RTK/β-arrestin interactions may be applicable to other GPCRs.

Many signalling effectors, such as Src, PI3K, ERK1/2 and MAPK can act as convergence points for multiple signalling pathways, including those that are GPCR or RTK mediated, making it more difficult to tease out signalling events directly attributable to transactivation. RTK inhibitors, such as AG1478 (EGFR), have been useful in ‘silencing’ the RTK component of transactivation; however, they often lack selectivity. Transactivation has largely been confirmed using indirect biochemical measures of signalling pathways (e.g. phosphorylation ERK1/2) at endogenous unmodified receptors. Although, they often lack dynamic, temporal or spatial resolution, these readouts can still reveal static spatial detail such as differential subcellular ERK1/2 and Akt activation in fractionated mice hearts and cardiomyocytes as a result of isoprenaline-induced βAR-mediated EGFR transactivation [30].

### The use of resonance energy transfer techniques to measure the real-time recruitment of adaptor proteins in transactivation

**Figure 2:**
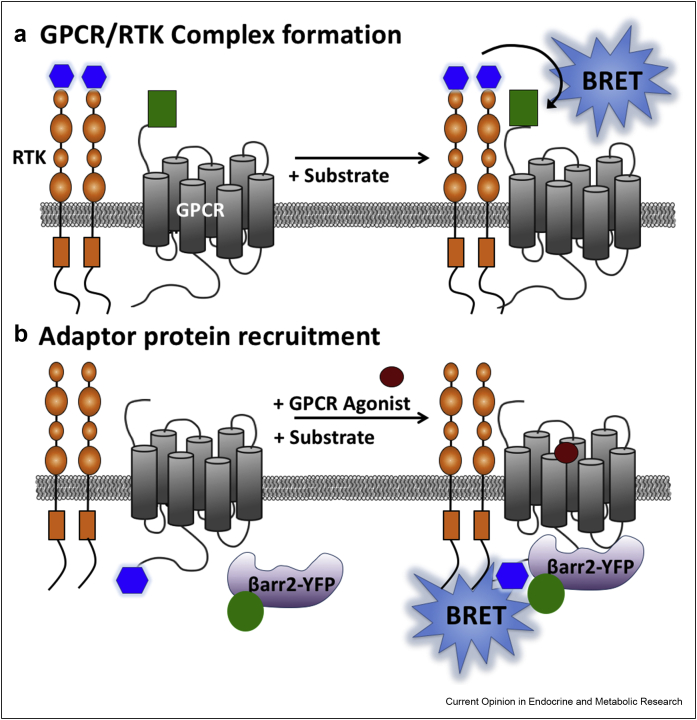
**Using bioluminescence resonance energy transfer to investigate GPCR/RTK complex formation and adaptor protein recruitment.** RTKs can be tagged at their N terminus with a luminescent protein (e.g. NanoLuc; termed the ‘donor’; *blue hexagon*) at both monomers, whereas a fluorescent tag (e.g. ‘SnapTag’ termed the ‘acceptor’; *green rectangle*) can be attached to the N terminus of a GPCR (**a**). The substrate for the luminescent protein is then oxidised, producing energy in the form of photons. If donor and acceptor tagged receptors are in sufficiently close proximity (<10 nm), non-radiative transfer of this energy occurs to excite the acceptor fluorophore. The ratio of fluorescence and luminescence emissions allows a BRET ratio to be determined. BRET can also be used to investigate adaptor protein recruitment to a GPCR/RTK complex (**b**), for example, using a GPCR tagged at its C terminus with a luminescence protein (*blue hexagon*) and a fluorescently tagged adaptor protein (in this case β-arrestin2-YFP; *green circle*).

**Figure 3:**
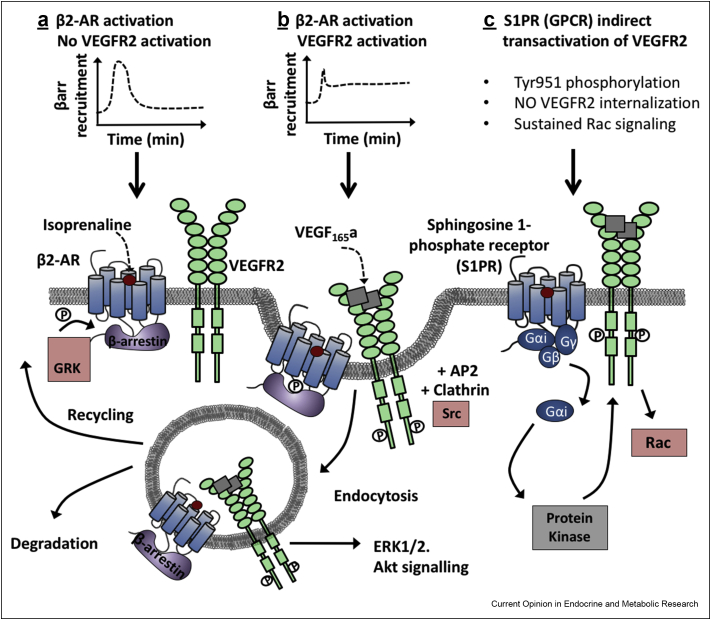
**Potential effects of transactivation of GPCR and vascular endothelial growth factor receptor 2 (VEGFR2) on effector protein recruitment or endocytosis.** Schematics (a) and (b) are based on findings detailed in Ref. [35] using BRET to measure β-arrestin2 recruitment to β2-adrenoceptor when co-expressed with the vascular endothelial growth factor receptor 2 (VEGFR2). β2-Adrenoceptors were stimulated with the agonist isoprenaline, resulting in GRK phosphorylation of the β2-adrenoceptor C terminus and subsequent rapid recruitment of β-arrestin2, which then subsided within minutes (a). However co-stimulated with isoprenaline and the VEGFR2 prototypical agonist VEGF_165_a altered the profile of β-arrestin2 recruitment (b). Although peak responses were truncated and then partially dropped, BRET signals did not return to baseline. This suggested that the presence of ligand-activated VEGFR2 lead to sustained β-arrestin2 coupling to β2-adrenoceptor. These data also reconciled with observations also seen in Ref. [35] that β2-adrenoceptors and VEGFR2 co-internalise into the same Rab5+ endosomal compartments following stimulation with either receptor agonist. This has the potential for modulation of signalling, in respect to pathway activation, kinetics of signalling or intracellular fate of receptors (recycling or degradation) when compared to endocytosis of either receptor alone. GPCRs have also been shown to indirectly modulate cell surface expression and endocytosis of RTKs resulting in altered signalling outcomes. The example depicted here is derived from data in Ref. [12] (c). Ligand-induced activation of a GPCR leads to Gαi-mediated activation of intracellular protein kinases, which can then directly phosphorylate specific tyrosine residues on the C terminus of the RTK. The example depicted here is in respect to sphingosine 1 phosphate receptor (S1P1 R)-mediated regulation of VEGFR2 endocytosis and involves the intracellular protein kinase c-Abl, phosphorylating VEGFR2 at tyrosine residue 951 (as opposed to the prototypical activation residue of Tyr1175) ultimately inhibiting VEGFR2 endocytosis. This leads to sustained VEGFR2-mediated Rac signalling that drives endothelial cell proliferation and enhances tumour growth.

There remains a need to quantify the real-time location and dynamics of transactivation specific signalling. Proximity-based techniques such as bioluminescence resonance energy transfer (BRET) or Förster Resonance Energy Transfer (FRET) offer exquisite spatial and temporal sensitivity for investigating protein–protein interactions in live cells due to the need for close proximity of donor/acceptor pairings (within 10 nm of each other) [31]. The use of a FRET-based biosensor illustrated the importance of phosphorylation by PI3K in regulating Src activity during transactivation of β2-AR/EGFR [32]. Furthermore, a BRET-based assay has highlighted the complexity of AT_1_R transactivation of insulin receptors as the protein kinase (ERK1/2 vs. PKC) mediator was found to differ between insulin receptor substrates [33]. BRET has also investigated the real-time kinetics of fluorescently tagged β-arrestin2 recruitment to β2-AR/IR complexes in response to isoprenaline [34] and at NanoLuc tagged β2-AR in the presence of VEGFR2 (Figure 2A [35]). The profile of β-arrestin2 recruitment to β2-AR was altered following agonist co-stimulation when compared to β2-AR agonist alone and required the presence of activated VEGFR2 (Figure 3A). In both cases β-arrestin was only seen with GPCR stimulation [34,35]. However Grb2 recruitment to AT_1_R/EGFR complexes measured using BRET revealed different extents depending on which receptor partner was activated, with rapid recruitment seen with EGF and only partial recruitment with the AT_1_R agonist angiotensin II [36]. Grb2 recruitment to AT_1_R/EGFR complexes in these cells (HEK293T) was shown to be independent of G_q/11_ or β-arrestin, whereas previous observations in COS-7 cells or ventricular cardiomyocytes showed dependence on G_q/11_ activation for AT_1_R/EGFR mediated hypertrophy [37]. These discrepancies may reflect differences in proximal (direct effector protein recruitment) versus indirect (e.g. downstream pathway activation) measures of transactivation [36].

However, it is also becoming increasingly clear that transactivation mechanisms may differ between cell types due to changes in the expression levels or repertoire of signalling components present. Functional genomic approaches (at AT_1_R/EGFR complexes in HMEC-LST cells [38]) and DNA microarray gene expression studies (α_2B_-AR in vascular smooth muscle cells [39]) have begun to provide new unbiased methods for identifying mediators involved.

## Physical complex formation between GPCRs and RTKs

The formation of oligomeric complexes between GPCRs and RTKs is now accepted as a regulator of transactivation [2]. These complexes may represent a mechanism to localise signalling components together to increase the efficiency of transactivation and resultant downstream signalling. Discrete complexes also raise the potential for cooperativity across putative GPCR/RTK interfaces; however, evidence for this is still largely speculative. Observations of complex formation have largely been derived from co-immunoprecipitation assays, which cannot definitively confirm physical complexes, their cellular location or lifespan. In contrast, the exquisite spatial sensitivity and dynamism of BRET and FRET techniques have recently been used to investigate GPCR or RTK oligomerisation in real time (reviewed in Ref. [31], summarised in Table 1).

**Table 1:** Summary of GPCR/RTK complexes detected using fluorescence- or luminescence-based techniques.

GPCR	RTK	Cell type	Technique used	Reference
5-HT1A	FGFR1	HEK293 cells (FRET, PLA), rat dorsal and median raphe nuclei (PLA)	FRET, PLA	[40]
5-HT1A	FGFR1	HEK293T cells, rat hippocampal cultures	PLA	[41]
5-HT1A	FGFR1	Rat brain dorsal hippocampus (astrocytes)	PLA	[45]
M1	FGFR1	Rat hippocampus and cerebral cortex	PLA	[46]
TSHR	IGF-1R	Graves orbital fibroblasts	PLA	[47]
β2-AR	IR	HEK293T cells	BRET	[42]
β2-AR	EGFR	HEK293T cells	FRET	[32]
AT_1_R	EGFR	HEK293T cells, CHO K1 cells, NIH-3T3, primary vascular smooth muscle cells	BRET	[36]
β2-AR	VEGFR2	HEK293 cells, HUVECs	BRET	[35]
Adenosine A_2A_	FGFR1	HEK293T cells	BRET	[43]
CB2R	HER2	HEK293T cells (BRET, bimolecular fluorescence complementation), Her2+ breast cancer patient biopsies (PLA)	BRET, PLA, bimolecular fluorescence complementation	[11]

BRET = bioluminescence resonance energy transfer.

FRET = fluorescence resonance energy transfer.

PLA = proximity ligation assay.

FRET has confirmed the formation of 5-hydroxytryptamine receptor 1 A (5-HT1A; GPCR) complexes with FGFR1 [40] supporting physiological evidence for these complexes and their role in neuronal plasticity [41]. BRET studies have also revealed the formation of heteromeric complexes between the β2-AR and IR that could underlie the counter-regulatory effects of insulin and catecholamines in glucose metabolism [42]. FRET has also shown isoprenaline-induced dissociation of β2-AR/EGFR complexes which internalise to distinct endocytic compartments [32]. Constitutive and dynamic agonist-induced complexes of AT_1_R/EGFR [36] and β2-AR/VEGFR2 [35] have also been revealed using BRET. β2-AR/VEGFR2 complexes, as measured by BRET, were also observed with endogenously expressed β2-AR (using CRISPR/Cas9 gene edited HEK293T cells) and in human umbilical vein endothelial cells (HUVECs [35]). Interestingly significantly increased BRET was observed between adenosine A_2A_ and FGF1 following concomitant agonist stimulation [43], consistent with previous biochemical observations in PC12 adrenal medulla cells where synergistic ERK1/2 phosphorylation was only observed with dual activation of A_2A_ and FGFR-1 [44]. Dissociation of complexes upon GPCR stimulation has also been observed with BRET studies of CB2R/HER2 in response to THC [11].

A disadvantage of RET-based studies is they cannot necessarily show the cellular location of GPCR/RTK complexes. Fluorescence imaging of co-localised GPCRs with RTKs has been limited by the paucity of selective antibodies for GPCR subtypes. The use of proximity ligation assays (PLA) has circumvented this in some ways with notable recent observations of endogenous heterocomplexes of 5-HT1A/FGFR-1 in rat hippocampal pyramidal neurons [40] and rat hippocampal astrocytes [45], muscarinic acetylcholine receptor 1 (M1)/FGFR1 complexes in hippocampal neurons [46], constitutive thyroid stimulating hormone receptor (TSHR) and IGF-1R in Graves orbital fibroblasts [47] and CB2R/HER2 complexes in HER2+ breast cancer patient biopsies [11]. Although PLA can provide improved spatial resolution, it is limited to use with fixed permeabilized cells and cannot reveal real-time changes. The use of genetically encoded fluorescent protein tags (e.g. GFP), or exogenously labelled tags (e.g. SnapTag or HaloTag) has allowed cellular co-localisation of GPCR/RTKs to be visualised both in absence or presence of ligands [35]. Questions remain as to whether changes in endocytosis of one partner may modulate transactivation. Stimulation with insulin can induce insulin receptor mediated internalisation of the β2-AR [42]. Insulin, acting via the IR, has been shown to stimulate internalisation of the β2-AR via IR-mediated phosphorylation of specific tyrosine residues in the β2-AR C terminus enhancing association with endocytosis components such as Grb2 [48]. Similarly, the β1 agonist dobutamine has been shown to induce partial internalisation of EGFR (β1-AR/EGFR [14]). The S1PR is able to promote VEGFR2 angiogenic signalling by regulating selective tyrosine phosphorylation of VEGFR2 via G_αi_ activation of the protein kinase c-Abl. Interestingly this retains VEGFR2 at the cell surface altering the kinetics of VEGFR2 driven Rac signalling from a transient to sustained profile. This results in increased migration of tumour-associated endothelial cells and ultimately tumour angiogenesis [12; Figure 3C]. Dual labelling of β2-AR/VEGFR2 revealed constitutive cell surface co-localisation [35]. Stimulation with receptor selective ligands resulted in co-endocytosis into early endosomal compartments which co-localised with immunolabelled Rab5 endosomes and reconciled with BRET data showing altered and sustained β-arrestin2 recruitment at these complexes (Figure 3B). This is interesting in light of the increasing appreciation of the importance of endosomal signalling in the spatiotemporal control of signalling for GPCRs [49] and RTKs [50].

RET and imaging studies have mostly used model cell systems due to the need to modify receptors with luminescent or fluorescent labels, which risk artefacts of receptor overexpression, although future use with CRISPR/Cas9 may mitigate this. In endogenous systems the extent of transactivation may be dependent on expression levels of each partner; however, interestingly BRET studies of angiotensin II-induced transactivation of EGFR by AT_1_R in HEK293T cells using overexpressed receptors revealed transactivation only represented a subset of the total signalling capacity of EGFR (estimated at ∼20% [36]). GPCR/RTK complex formation may therefore represent a subset of RTKs arranged in membrane microdomains or within intracellular compartments that facilitate close proximity and the formation of discrete complexes with their partner GPCR. Advanced imaging techniques with single cell/receptor sensitivity such as fluorescence correlation spectroscopy (FCS) and total internal reflection fluorescence microscopy (TIRF-M) have illustrated that receptors are not homogenously expressed on the surface of cells, but are within discrete membrane regions [51]. This localises components of signalling within microdomains, bringing different signalling mediators into close proximity, facilitating greater efficiency of receptor/effector coupling. These specialised microdomains termed ‘lipid rafts’ are linked to the actin cytoskeleton [52]. Signalling as a consequence of transactivation of CBR1/FGFR1 complexes has been shown to emanate from lipid rafts in embryonic cortical neurons [53]. As many GPCRs and RTKs are known to localise to caveolin containing lipid rafts [54], it is likely that other GPCR/RTK complexes may also exist here.

Unravelling GPCR/RTK complex formation is further complicated by RTK heterodimerisation. Recent FRET studies have indicated that RTK homo and heterodimers have similar strength of interactions, highlighting the potential influence that RTK heterodimer may have upon transactivation [55]. The increasing acceptance of GPCR homo and heteromerisation (albeit likely to be relatively transient) may also further complicate understanding of transactivation signalling networks [56]. GPCR/RTK complexes may also be components of larger macromolecular complexes containing other membrane bound proteins such as integrins, extracellular matrix glycoproteins and co-receptors (e.g. Neuropilin-1 for VEGFR2 [57]). Investigation of the influence of these proteins on GPCR/RTK complex formation, organisation, lifetime and signalling is still in its infancy. The altered signalling seen with GPCR/RTK transactivation suggests that co-targeting of GPCR/RTK macromolecular complexes may represent new therapeutic avenues; wholesale inhibition of RTK signalling can often result in considerable off-target effects due to the integral role RTKs play in physiological processes. The use of lower concentrations of RTK inhibitors in conjunction with ‘trans-inhibition’ of GPCR partners may provide a mechanism to modulate RTK-driven signalling to overcome some of these off-target issues.

## Conclusion

GPCRs have been shown to exploit the intrinsic kinase activity and vast signalling networks available to RTKs, whereas proteins previously defined as ‘GPCR signalling mediators’ are now known to also be integral signalling partners for RTKs (e.g. G proteins, β-arrestins). This bidirectional transactivation between GPCRs and RTKs allows integration of signalling inputs to increase the number and diversity of signalling outcomes available. The advancement of fluorescence- and luminescence-based techniques has allowed the identification of GPCR/RTK complexes whose dynamics, localisation and distinct pharmacological profiles can be quantified in real time. Studies of cooperativity across GPCR and RTK interfaces are still relatively understudied; however, advancements in techniques that offer increased real-time spatial and temporal resolution will allow this phenomenon to be teased apart from signalling crosstalk and may open up new opportunities to co-target GPCR/RTK complexes in drug discovery.

## Conflict of interest statement

Nothing declared.
